# The origin of multiple clones in the parthenogenetic lizard species *Darevskia rostombekowi*

**DOI:** 10.1371/journal.pone.0185161

**Published:** 2017-09-20

**Authors:** Alexey P. Ryskov, Fedor A. Osipov, Andrey V. Omelchenko, Seraphima K. Semyenova, Anastasiya E. Girnyk, Vitaly I. Korchagin, Andrey A. Vergun, Robert W. Murphy

**Affiliations:** 1 Laboratory of Genome Organization, Institute of Gene Biology of the Russian Academy of Sciences, Moscow, Russia; 2 Department of Biochemistry, Molecular biology and Genetics, Moscow State Pedagogical University, Moscow, Russia; 3 Group of Bioinformatics and Modeling Biological Process, Severtsov Institute of Ecology and Evolution of the Russian Academy of Sciences, Moscow, Russia; 4 Department of Natural History, Royal Ontario Museum, Toronto, Ontario, Canada; National Cheng Kung University, TAIWAN

## Abstract

The all-female Caucasian rock lizard *Darevskia rostombekowi* and other unisexual species of this genus reproduce normally via true parthenogenesis. Typically, diploid parthenogenetic reptiles exhibit some amount of clonal diversity. However, allozyme data from *D*. *rostombekowi* have suggested that this species consists of a single clone. Herein, we test this hypothesis by evaluating variation at three variable microsatellite loci for 42 specimens of *D*. *rostombekowi* from four populations in Armenia. Analyses based on single nucleotide polymorphisms of each locus reveal five genotypes or presumptive clones in this species. All individuals are heterozygous at the loci. The major clone occurs in 24 individuals and involves three populations. Four rare clones involve one or several individuals from one or two populations. Most variation owes to parent-specific single nucleotide polymorphisms, which occur as heterozygotes. This result fails to reject the hypothesis of a single hybridization founder event that resulted in the initial formation of one major clone. The other clones appear to have originated via post-formation microsatellite mutations of the major clone.

## Introduction

Questions concerning origin and evolution of parthenogenesis in lizards have received considerable attention in recent years [1–6]. Parthenogenetic lizard species are ideal organisms for studying the genetic and ecological basis of hybridogeneous speciation, understanding the mechanisms of hybrid parthenogenesis, generation and evolution of genetic diversity, determination of possible factors of natural selection, and hybrid disfunction caused by genetic incompatibilities of admixed genomes or re-established balance of cytonuclear genomes. Unisexual species have been found in less than 0.1% of all vertebrate species [2].

Among vertebrates, true parthenogenesis, where reproduction proceeds without male participation, has been detected in squamate reptiles, especially in lizards [7] and also in the brahminy blind snake *Indotyphlops braminus* [8,9]. It also occurs in other vertebrate groups, e.g. the bonnethead shark *Sphyrna tiburo* [10]. The Lacertidae was the first family in which this phenomenon was discovered [11]. In most known instances, parthenogenetic species originated from interspecific hybridization between closely related bisexual species [12,13], but these may not be sister-species [14]. Only parthenogenetic species of *Lepidophyma* (*L*. *reticulatum* and *L*. *flavimaculatum*) [2] and the parthenogenetic triploid species *Leposoma percarinatum* [15] did not possibly originate from hybridization between bisexual species. (Further studies are necessary in these exceptional cases). The hypothesis of hybrid origin postulates that two species interbred and produced viable offspring that then reproduced clonally. According to the balance hypothesis [16], a new successful, clonal species can be established if genetic differences between the hybridizing species are sufficient to disrupt recombination during meiosis and while not severely limiting viability, fecundity, and other characteristics affecting fitness of the hybrid species. In other words, hybridization is dependent on phylogenetic distance. In contrast, the phylogenetic constraint hypothesis of Darevsky (1967) states that hybridization is idiosyncratic (linage dependent) [17]. Because of their hybrid origin, parthenogenetic species combine much of the genetic diversity of two parental sexual species. They are normally diploid (as in *Darevskia*) or triploid (as in *Aspidoscelis*) and commonly exhibit high levels of nuclear gene diversity because of fixed heterozygosity at codominant loci [18]. The more divergent hybridizing parental species are, the greater ability to persist in nature over many generation because distinct parental alleles form heterozygous loci in the hybrid genome. Sister-chromatid pairing provides a mechanism for maintaining heterozygosity, which is critical for offsetting the reduced fitness in parthenogenetic species [19].

Each parthenogenetic species usually consists of several clonal lineages, possibly caused by either mutations (especially in hypervariable microsatellite loci), multiple origins from different pairs of founders, rare new hybridization events, or some level of genetic recombination [16,20,21]. Clonal diversity also appears to correlate with the size of the area of origin, distinct ecological conditions of the habitat, and age of parthenogenetic species [22–24]. Based on patterns of allozyme variation observed among parthenogenetic species of a number lizard genera, Parker et al. proposed a model to predict whether they arose from single or multiple interspecific hybridization events [23]. Accordingly, a species with a single hybridization origin typically has little allozyme and mitochondrial DNA (mtDNA) variation and consists of a widespread major clone with a few rare subclones. In contrast, a species with multiple hybridization origins has a highly variable pattern of genetic variation, with seemingly random combinations of alleles. However, the situation can be more complex. For example, high levels of allozyme variation with low level of mtDNA variation can suggest multiple hybridization origins, such as for *Cnemidophorus lemniscatus* [25,26] and *Heteronotia binoei* [27]. *Aspidoscelis neomexicana* [28] and *A*. *tesselatus* [25] exhibit low levels of allozyme and mtDNA variation yet each species has several geographically restricted origins. Thus, different mutually non-exclusive models might apply to the origin of clonal lineages. Future effort should investigate the clonal diversity in various parthenogenetic species.

The lacertid genus *Darevskia* is of particular significance because its species have been the subjects of extensive ecological and biogeographical study, and because parthenogenesis has arisen several times within the group [12]. *Darevskia* includes 24 bisexual species and seven parthenogenetic, diploid species of hybrid origin [29]. Previous studies on these parthenogenetic species revealed some degree of allozyme variation and low variability of mitochondrial DNA [24,30–34]. Generally, the allozyme patterns in the parthenogenetic *Darevskia* are in accordance with Parker et al.’s model [23], with the exception of *D*. *rostombekowi*.

*Darevskia rostombekowi* (spelling following Murphy [35]) is one of the seven parthenogenetic species that arose from the interspecific hybridization of *D*. *portschinskii* (paternal species) and *D*. *raddei* (maternal species). The parental species belong to different clades of *Darevskia* [13,24] and the formation of parthenoforms appears to be phylogenetically constrained [14]. Phylogenetic factors also restrict the formation of some other parthenogenetic species [4]. *Darevskia rostombekowi* have a chromosome set of 2*n* = 38 [36], are characterized by fixed heterozygosity of allozyme loci [34] and exhibit low variability of restriction sites of mitochondrial DNA inherited from *D*. *raddei* [24]. Studies of 35 allozyme loci from populations in northwestern and central Armenia revealed no allozyme variability, which suggested that this species could be monoclonal [30]. This was unlike the parthenogenetic species *D*. *dahli*, *D*. *armeniaca*, and *D*. *unisexualis*, in which several allozyme clones were found [31]. At present, the paternal species *D*. *portschinskii* occurs in southern Georgia, northern Armenia and western Azerbaijan, and the maternal species *D*. *raddei* is distributed in Armenia and adjoining Georgia, western Azerbaijan, and northern/western Iran. Several discrete populations of *D*. *rostombekowi* occur in Lori and Tavush provinces of northern Armenia (elevation range 700–1500 m), and one small, isolated high-montane (2000 m) population along the southeastern shore of Lake Sevan (Gegharkunik province), in the vicinity of the village of Zagalu (renamed recently to Tsovak). The Sevan and Vardenis mountain ranges, which rise to more than 3000 m, isolate this population from the nearest populations inhabiting the foothills near the Kura River by Lake Gai-Gel’, Azerbaijan. The mountains are insurmountable geographical barriers for *D*. *rostombekowi*. No allozyme analysis exists for this population.

Analysis of mitochondrial DNA (mtDNA) showed that all northern matrilines of Armenian *D*. *rostombekowi* (Papanino, Gosh, Spitak) originated from one southern Armenian population (Yeghegnadzor) of *D*. *raddei* [24]. Initial analyses of mtDNA (cytochrome *b*; *Cytb*) from *D*. *rostombekowi* at Tsovak indicated a maternal origin from *D*. *raddei* at Yeghegnadzor [37], but subsequent study based on other mtDNA and nuclear DNA markers [38–40] resolved a single origin. All specimens from Tsovak had a single nucleotide substitution in *Cytb*, which suggested the existence of a second matriline of *D*. *rostombekowi* specific to Tsovak. In addition, interpopulation comparisons of genetic variation in *D*. *rostombekowi*, based on multilocus DNA fingerprinting with microsatellite hybridization probes, and RAPD markers for the nuclear genome, indicated significant differences between lizards from northern Armenian and Tsovak [41,42]. These findings, plus considerable morphological variation observed among lizards from different populations [13], highlight new questions concerning intra- and inter-population genetic variation and clonal diversity.

Our study examines the clonal diversity in *D*. *rostombekowi* including variation at three microsatellite-containing loci identifies genotypes of *D*. *rostombekowi* from four Armenian populations. We apply a new approach to genotyping the related parthenogenetic species *D*. *dahli* [43] and show that *D*. *rostombekowi*—previously presumed to be a monoclonal parthenogenetic species—has several clones. New, the approach reveals parent-specific markers consisting of microsatellites and single nucleotide polymorphisms (SNPs) located out of the microsatellite cluster for each allele of each locus. The SNPs yield direct information about interspecific hybridization founder events, and microsatellite variability provides information concerning possible mutations in the initial hybrid clones that give rise to new genotypes. Interspecies allele comparisons involve homologous microsatellite loci from the parental bisexual lizard species *D*. *raddei* and *D*. *portschinskii*. All alleles present in *D*. *rostombekowi* also occur in the parental species.

## Materials and methods

**Fig 1:**
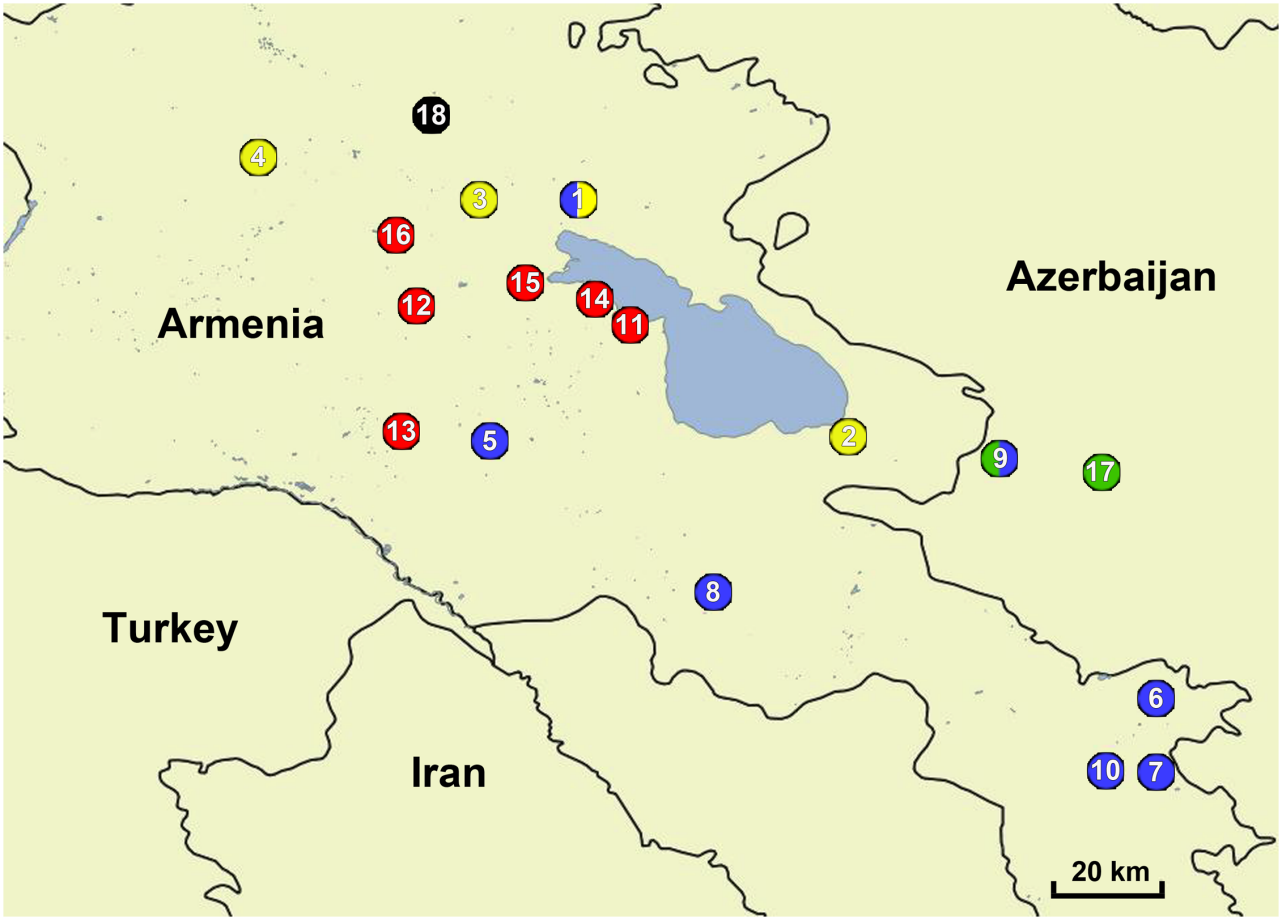
Map of Armenia showing the localities from which populations of parthenogenetic *Darevskia rostombekowi* and bisexual parental species *D*. *raddei* and *D*. *portschinskii* were collected. Sampling localities are indicated by the following colors: *D*. *rostombekowi*–yellow; *D*. *raddei raddei*–blue; *D*. *raddei nairensis*–red; *D*. *portschinskii portschinskii*–green; *D*. *portschinskii nigrita*–black. Numbers indicate populations: 1 –Gosh (40°42'20.3"N 45°00'57.7"E); 2 –Tsovak (40°10'45.0"N 45°37'22.7"E); 3 –Papanino (40°42'27.7"N 44°45'43.8"E); 4 –Spitak (40°48'50.0"N 44°16'48.7"E); 5 –Geghard (40°08'49.4"N 44°48'26.9"E); 6 –Goris (39°33'09.5"N 46°21'19.7"E); 7 –Doroga (39°22'53.9"N 46°21'06.6"E); 8 –Yeghegnadzor (39°47'48.4"N 45°19'52.4"E); 9 –Kelbajar (40°06'03.1"N 45°59'27.1"E); 10 –Tatev (39°23'13.2"N 46°15'11.2"E); 11 –Ayrivank (40°26'02.3"N 45°06'27.2"E); 12 –Bjni (40°27'42.6"N 44°39'07.3"E); 13 –Yerevan (40°10'37.0"N 44°36'09.3"E); 14 –Lchap (40°28'02.4"N 45°03'43.5"E); 15 –Lchashen (40°30'45.9"N 44°54'03.2"E); 16 –Pyunik (40°36'49.9"N 44°35'06.4"E); 17 –Zuar (40°04'39.0"N 46°13'47.0"E); 18 –Dzoraget (40°54'15.0"N 44°40'37.7"E).

DNA samples of *D*. *rostombekowi* (n = 42) from four populations, *D*. *raddei* (n = 65) from 13 populations, and *D*. *portschinskii* (n = 27) from two populations in Armenia were analyzed (Table 1). Sample localities were shown in Fig 1. All DNA was extracted from samples obtained about 15 years ago. Parthenogenetic and bisexual lizards were collected between 1997 and 2006 from their natural habitats in Armenia. All collecting sites occurred on governmental land that did not involve protected areas. Therefore, specific permissions were not required for collecting in these locations. Our field studies predated the listing of *D*. *rostombekowi* as an Endangered Species by the IUCN (2009) and the species is not protected by CITES. The study was approved by the Ethics Committee of Moscow State University (Permit Number: 24–01) and was carried out in strict accordance with their ethical principles and scientific standards. All specimens were captured with a noose. Blood samples were taken from the tail veins of lizards under chloroform anesthesia, and then these lizards were released. DNA was isolated from lizard blood by using the standard phenol–chloroform extraction method with proteinase K, and resuspended in TE buffer, pH 8.0.

**Table 1:** Species and population samples used in this study.

Species	Populations	Number of individuals in populations	Total number of species individuals
*D*. *rostombekowi*	Gosh	4	42
	Tsovak	8	
	Papanino	21	
	Spitak	9	
*D*. *raddei raddei*	Yeghegnadzor	8	20
	Geghard	3	
	Gosh	1	
	Kelbajar	1	
	Tatev	3	
	Doroga	2	
	Goris	2	
*D*. *raddei nairensis*	Pyunik	17	45
	Lchap	5	
	Lchashen	14	
	Yerevan	6	
	Bjni	1	
	Ayrivank	2	
*D*. *portschinskii portschinskii*	Zuar	15	15
*D*. *portschinskii nigrita*	Dzoraget	12	12

Three loci, Du215, Du281, and Du323 were PCR-amplified using previously described primer pairs [43,44]. These loci were chosen because each contained a tetranucleotide microsatellite cluster that provided sufficient resolution of the individual allelic PCR products in non-denaturing polyacrylamide gel electrophoresis to allow the direct sequencing of the individual alleles. In general, the procedure for the isolation and sequencing of individual alleles was carried out as described previously [44,45].

Data on genetic variation of these loci in *D*. *rostombekowi* [46] and on allelic variation of the homologous loci in *D*. *raddei* and *D*. *portschinskii* were shown in S1 Table. PCR was performed on 50 ng of DNA in a total volume of 20 μl using a GenePak PCR Core Kit (Isogene) and 1 μM of each primer. The reaction conditions were as follows: one cycle of 3 min at 94°C; 30 cycles of 1 min at 94°C; 40s at the annealing temperature (58°C for Du215, 50°C for Du281, and 48°C for Du323); and 40s at 72°C followed by one cycle of 5 min at 72°C. PCR products (15 μl) were loaded onto an 8% non-denatured polyacrylamide gel (to separate allelic variants for each locus) and run for 12 h at 60V. A 50bp ladder (Fermentas) was used as a size marker. The amplified products were visualized by staining DNA in the gel with ethidium bromide. Well-resolved individual PCR products, which corresponded to the two individual alleles of the locus, were excised from the gel, purified by ethanol precipitation, and sequenced directly in both directions using a chain termination reaction with an ABI PRISM BigDye Terminator v.3.1 on an Applied Biosystems 3730 DNA analyzer. Allelic identity was checked and confirmed via the comparison of sequences obtained independently. All unique de novo sequences were deposited in GenBank (KM573728–KM573762; HM014002–HM014003; KR559279–KR559316).

The number of alleles, allelic richness, as a measure of allele counts adjusted for sample size, and expected heterozygosity, as a measure of gene diversity, were calculated per locus and per population by using Fstat v.2.9.3.2 [47], GenePop v.4.2, and Web-version of POPTREEW [48]. Analysis of molecular variance AMOVA was carried out using the package poppr [49]. We implemented multiple alignments of microsatellite allele sequences obtained from the three species by using MUSCLE [50]. These matrices were used for the building a neighbor joining network with MEGA v.6.0.6 [51]. The method of coding genotypes was described previously [45].

## Results

All individuals of *D*. *rostombekowi* were heterozygous at the three loci and contained two alleles that differed from each other in length and structure of the microsatellite clusters. Two of them, Du281 and Du323, had also single nucleotide variants (SNVs) in fixed positions of the flanking regions (S1 Table). Unlike SNPs in sexual species, SNVs in hybrid genomes of parthenogenetic lizards result from the codominant loci of divergent parental genomes. They represent parent-specific markers in parthenogenetic species. Thus, distinct clonal SNVs would likely have owed to independent hybridization founder events.

The number of alleles in *D*. *rostombekowi* varied from two to five depending on the locus (S1 Table). The allelic variants of Du281 and Du 323 formed distinct groups according to parent-specific SNVs. SNVs in alleles 1–3 of Du281 formed the SNV set TATA, whereas those in allele 4 of Du281 formed the set CGCG. Similarly, SNVs in alleles 1 and 2 of Du323 had two sets of SNVs: CT and AC. These variants reflected the hybrid origins of the parthenogenetic species and the heterozygosity of its genome. Du215 did not possess parent-specific SNVs in the vicinity of the microsatellite cluster (S1 Table). The dinucleotide insertion TT (-98/99) Du215(rost)3 occurred only in one individual from Gosh and this insertion was not found in those populations of the parental species analyzed (see below and S1 Table). In *D*. *rostombekowi* (n = 42), Du215 had five alleles, Du281 had four, and Du323 had two (S1 Table).

The origin of SNVs at Du281 was shown schematically in S1 Fig based on alignment of the microsatellite sequences and neighboring regions. The SNVs of Du281 connected with parental alleles at the heterozygous locus.

To identify genotypic diversity, we constructed allelic combinations in the 42 individuals of *D*. *rostombekowi*. Based on both single nucleotide and microsatellite variants, analyses resolved five genotypes that differed in their frequencies and geographic distribution (Table 2).

**Table 2:** Sample size, genotype composition, diversity and distribution in the populations of D. *rostombekowi*.

Genotype number	Genotype composition (see, Fig 2)	Population				Number of individuals with definite genotype (genotype frequencies)
		Gosh	Tsovak	Papanino	Spitak	
1	Du215(2+5)+Du281(1+4)+Du323(1+2)	2	0	14	8	24 (0.571)
2	Du215(2+5)+Du281(2+4)+Du323(1+2)	0	0	7	1	8 (0.192)
3	Du215(4+5)+Du281(3+4)+Du323(1+2)	0	8	0	0	8 (0.189)
4	Du215(1+5)+Du281(1+4)+Du323(1+2)	1	0	0	0	1 (0.024)
5	Du215(3+5)+Du281(2+4)+Du323(1+2)	1	0	0	0	1 (0.024)
Total number of individuals		4	8	21	9	42
Genotype diversity (%)		3 (75.0)	1 (0)	2 (9.5)	2 (22.2)	5

Individuals with identical genotypes were assumed to represent distinct clonal lineages. Occurring in 24 individuals (57.1% of the total cohort), genotype 1 was most abundant; it was found in three populations. Genotypes 2 and 3 were represented by eight individuals each (19% of the total cohort) in two and one population, respectively. Finally, rare genotypes 4 and 5 were represented by one individual each (2.38% of the total cohort).

The genotypic diversity among the four populations of *D*. *rostombekowi* varied from 0% to 75% (Table 2). The highest level of genotypic diversity was observed at Gosh, where rare genotypes 4 and 5 occurred; both variants might have originated via post-formation microsatellite mutations from the most abundant, and presumptively initial, genotype 1. The lowest level of genotypic diversity was observed at Tsovak, which had genotype 3 only. This genotype differentiated remote Tsovak from all populations in northern Armenia. We carried out AMOVA of four *D*. *rostombekowi* populations. However, the results of AMOVA were insignificant probably because of low sample size in most of the localities. Population genetic indices for k loci and genotypes 1–5 were given in Table 3. Expected heterozygosity varied from 0.500 to 0.750 (average, 0.534) and the number of alleles ranged from two to four (average, 2.7). Values of allelic richness ranged from 1.995 to 4.000 (average, 2.620). The highest allelic scores, number of alleles and allelic richness occurred at Gosh for the all three loci.

**Table 3:** The population indices of gene diversity for three studied loci in four sampled populations of *D*. *rostombekowi*.

Locus	Population	Allele (N)	R_s_	H_E_
Du215	Gosh	4	4.000	0.750
	Zagalu	2	2.000	0.533
	Papanino	2	1.997	0.512
	Spitak	2	2.000	0.533
	∑	5	2.738	0.520
Du281	Gosh	3	3.000	0.679
	Zagalu	2	2.000	0.533
	Papanino	3	2.773	0.626
	Spitak	3	2.443	0.533
	∑	4	3.127	0.582
Du323	Gosh	2	2.000	0.571
	Zagalu	2	2.000	0.533
	Papanino	2	1.997	0.512
	Spitak	2	2.000	0.533
	∑	2	1.995	0.500
All loci		11	2.620	0.534

N—number of alleles, R_s_—allelic richness, H_E_—expected heterozygosity.

Indices of gene diversity in males and females of the parental species *D*. *raddei* (n = 65) and *D*. *portschinskii* (n = 27) were given in Table 4. Their allelic diversity was higher than that of the parthenogenetic populations. Du323 had lowest number of alleles, ranging from 2 to 7, depending on species. The number of alleles for Du281 varied from 8 to 15, and for Du215 from 9 to 17, depending on species. The values of allelic richness at all three loci were equal for males and females in *D*. *raddei*, and a little more for male *D*. *portschinskii*. Du215 and Du281 had heterozygote deficits. That of Du281 also occurred in male *D*. *raddei*. Female *D*. *raddei* had an excess of heterozygotes at Du323. Deviations from Hardy-Weinberg expectations probably owed to small sample sizes. The heterozygote deficits may have owed to a Wahlund effect due to the 65 individuals of *D*. *raddei* coming from 13 localities.

**Table 4:** The generalized indices of gene diversity for three studied loci in males and females of sampled populations of bisexual lizards *D*. *raddei (N = 65) and D*. *portschinskii (N = 27)*.

Species	Locus	Allele (N)	R_s_	H_E_	Global Hardy-Weinberg test when H1 = heterozygote deficit (P)	Global Hardy-Weinberg test when H1 = heterozygote excess (P)
*♂♂D*. *raddei* (N = 38)	Du215	14	1.694	0.4932	0.8286	0.1866
	Du281	15	1.917	0.8761	**0.0057**	0.9957
	Du323	2	1.447	0.3378	1.0000	**0.0001**
	All loci	31	1.686	0.5690	0.6861	0.3149
*♂♂D*. *portschinskii* (N = 21)	Du215	17	1.925	0.8831	**0.0000**	1.0000
	Du281	8	1.841	0.7149	**0.0002**	1.0000
	Du323	7	1.827	0.6795	0.1844	0.7999
	All loci	32	1.864	0.7591	**0.0000**	1.0000
*♀♀D*. *raddei* (N = 27)	Du215	9	1.597	0.3867	0.5208	0.5070
	Du281	12	1.892	0.8773	0.3192	0.6644
	Du323	2	1.475	0.3800	1.0000	**0.0001**
	All loci	23	1.654	0.5480	0.9631	0.0400

**Fig 2:**
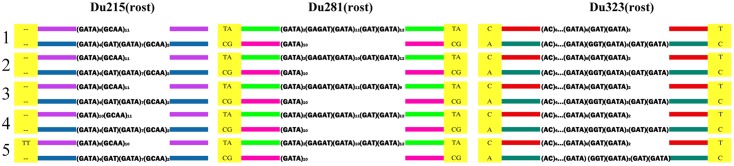
Schematic representation of five genotypes formed by allelic combinations of microsatellite loci Du215, Du281, and Du323 in 42 individuals of *Darevskia rostombekowi*. Parent-specific SNV markers are shown in yellow squares. Variable microsatellite clusters are shown in each of two alleles.

To determine if *D*. *rostombekowi* originated from a single or multiple interspecies hybridization event(s), genotype-specific markers, formed by combinations of parent-specific SNVs of both alleles in Du281 and Du323 were evaluated (Fig 2; S1 Table). All genotypes matched the parent-specific combinations TATA/CGCG (Du281) and CT/AC (Du323). This suggested a common origin of all five genotypes owing to a single interspecies hybridization event. However, we could not rule out the possibility of independent crossings of parental individuals possessing the same alleles in these loci. Genotype 5 in one individual from Gosh had the same genotype-specific markers for loci Du281 and Du323, but differed in having unique allele Du215(rost3) with its dinucleotide insertion TT(-98/99). This insertion was not detected among analyzed parental alleles (see below; S1 Table) and, thus, it probably represented a *de novo* mutation.

Genotypes 1–5 differed from each other by microsatellite sequences only. Some of the rarer genotypes may have arisen through post-formation microsatellite mutations of the dominant genotype. Genotype 3 from Tsovak was the most divergent. Genotypes 4 and 5 appeared to be independent events and they were observed only in the Gosh population, which had the most variability of genotypes. No unique genotypes were found at Papanino and Spitak. The analysis of the spatial-frequency distributions of the more widely distributed genotypes 1 and 2, and population-specific genotype 3 revealed a dependence between the frequencies of the clones and the geographical disjunction between the three northern populations and the Tsovak population.

**Fig 3:**
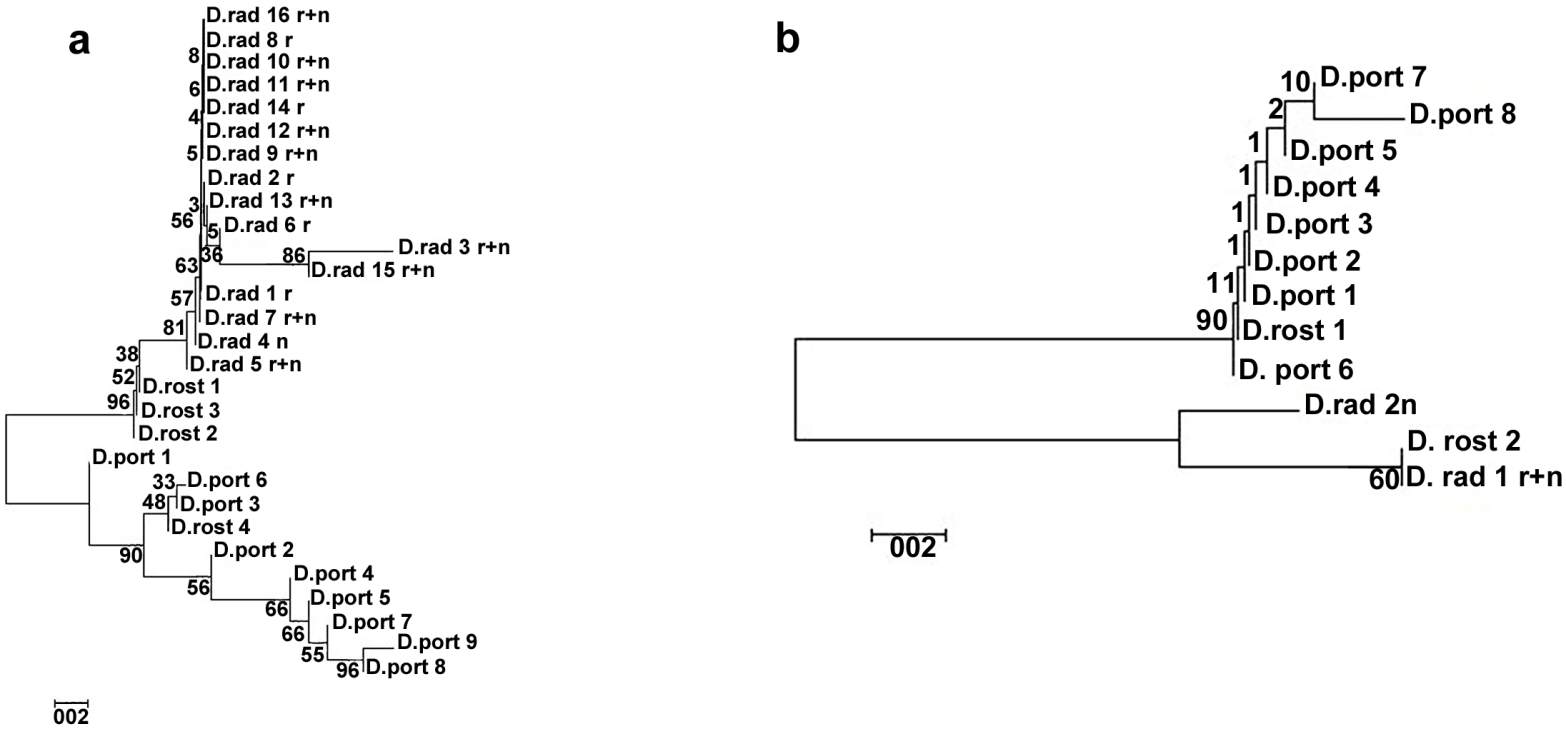
Genetic similarity between the sequences of alleles Du281 (a) and Du323 (b) for the species *D*. *portschinskii* (D. port), *D*. *rostombekowi* (D. rost) and *D*. *raddei* (D. rad). NJ/p-distance/Pairwise deletions/IB = 500.

Alleles of Du215, Du281, and Du323 in *D*. *rostombekowi* were compared with those of *D*. *raddei* (n = 65) and *D*. *portschinskii* (n = 27). Sequencing obtained 37 alleles from *D*. *raddei* and 38 from *D*. *portschinskii* (S1 Table). Microsatellite motifs and the adjoining nucleotides were used to discern parental alleles inherited by *D*. *rostombekowi*. Among 19 alleles for Du215, 16 for Du281, and two for Du323 in *D*. *raddei*, *D*. *rostombekowi* possessed two only: Du323(rost)2 formed Du323(rad)1 in *D*. *raddei* and allele Du323(rost)1 occurred in *D*. *portschinskii* as Du323(port)5. Similarly, alleles Du215(rost)1–4 were the same as Du215(port)7, 5, 10, 7, respectively, and Du215(rost)5 was the same as Du215(rad)12. Further, Du281(rost)1–3 had the motif (GATA)_n_, and specifically (GATA)_2_ GAGAT. The locus also appeared as Du281(rad)3 and Du281(rost)4 with a perfect (GATA)_10_; these coincided with alleles Du281(port)6 and 7, respectively. Due to the high mutation rate of microsatellite DNAs [45], in some cases we did not find direct correlations between distinct alleles of *D*. *rostombekowi* and its parents. The dendrograms of genetic similarity for Du281 and Du323 loci, for which we found SNV markers, were shown in Fig 3a and 3b. Both bisexual species formed distinct clusters and some alleles of the parthenogens were inherited from maternal species *D*. *raddei*, and others from paternal species *D*. *portschinskii*. For example, Du281(rost)1, 2, and 3 showed similarity with all Du281 alleles of *D*. *raddei*, and Du281(rost)4 was similar to two Du281 alleles of *D*. *portschinskii*. Du323(rost)1 had high similarity with Du323 alleles of *D*. *portschinskii*, and Du 323(rost)2 had high similarity with Du323 alleles of *D*. *raddei*.

## Discussion

Given that lizards with identical genotypes represent a distinct clone, the several microsatellite clones of *D*. *rostombekowi* reject the hypothesis of monoclonality based on allozymes [30]. However, it corresponds with the considerable amount of morphological variation in this species [13]. The observed difference in patterns of allozymes and microsatellites likely reflects the less variable nature of allozymes compared with microsatellites. Parthenogenetic *D*. *armeniaca*, *D*. *dahli* and *D*. *unisexualis* have more than one allozyme clone [52] and microsatellites may reveal additional clones. Our approach, which is based on detection of both, microsatellite variability and single nucleotide variation of DNA sequences outside of microsatellite clusters, promises to reveal even higher levels of clonal diversity in these species. For example, an analysis of 35 allozyme loci revealed five clones in *D*. *dahli*, whereas three microsatellite-containing loci detected 11 clones [43]. Identical genotype-specific markers occur all clones of *D*. *rostombekowi*, suggesting that it was formed by one hybridization event. Identical SNVs but different microsatellite markers show that the hybridization event involved very similar female *D*. *raddei* and male *D*. *portschinskii* in one population.

It remains unclear which of these clones of *D*. *rostombekowi* formed first. Assuming that unisexual species originating from a single hybridization exhibit a widespread and common clone with a few rare clones [23], genotype 1 of *D*. *rostombekowi* might be ancestral (Table 2). The other clones likely represent post-formation microsatellite mutations. The highly unstable (GATA)_n_-containing locus in parthenogenetic *D*. *unisexualis* appears to have accumulated mutant alleles in some offspring of the first generation; *de novo* mutations occur via deletion or insertion of a single microsatellite repeat [45]. Such mutations might be responsible for the origin of genotypes 4 and 5 of *D*. *rostombekowi* at Gosh and genotype 2 at Papanino and Spitak (Table 2). Surprisingly, major genotype 1 and genotypes 2, 4, and 5 remain undetected at Tsovak. This discovery questions the origin of genotype 3. Genotype 3 may owe to an independent hybridization event. However, we do not have a sufficient number of genotype-specific markers to test for this possibility. Alternatively, genotype 3 may have originated from common genotype 1, only to completely replace it. Ecological, physical and climatic differences among the habitats of various populations of *D*. *rostombekowi* may probably explain interpopulational differences in the distribution of various microsatellite genotypes [53–55]. Diversification may also relate to the frequency of interspecific mating at different localities, the relative fitness of clonal lineages and/or outcompeting hybrids its sexual progenitors [56].

Analysis of mitochondrial DNA shows that *D*. *raddei* from Yeghegnadzor is the matriarch of all northern populations (Papanino, Gosh, Spitak). However, the finding of a second mitotype of *D*. *rostombekowi* specific for the Tsovak population [37] indicates the possible participation of different females of *D*. *raddei* in the hybridization. To examine this possibility, additional genotype-specific markers from more microsatellite loci are necessary.

Parthenogenetic species of *Darevskia* exhibit low levels of clonal diversity and a high degree of mtDNA similarity with respect to their parental species. Their recent origins appear to best explain these patterns, while they may have largely different ages. Darevsky [57] suggested that all parthenogenetic species of *Darevskia* originated in the Caucasus after the last glacial maximum and that all species have had the same amount of time to evolve new clones, approximately 5000–7000 years ago. However, a multilocus analysis for the parthenogens *D*. *unisexualis*, *D*. *bendimahiensis* and *D*. *uzzeli* dated their origins to between 200 000 and 70 000 years ago [6]. These results further support the independent origins of parthenogenesis in *Darevskia* and these predate the last glacial maximum. Different parental lineages hybridized several times in different geographic regions [14]. Further, Irwin et al. [58] provided an estimate of a divergence rate of approximately 10% per million years for the silent substitution at the third-codon position, based on mammalian *Cytb* data. If this calculation is applicable to lizards, the age of *D*. *rostombekowi* would be approximately 200 000 years old (2% divergence) [59].

Electrophoretic studies of proteins and mitochondrial DNA have shown that parthenogenetic lizards tend to be multiclonal. A high level of variability of allozyme loci was discovered in *Heteronotia binoei* (Gekkonidae) [16]. Genetic heterogeneity was also demonstrated for the American parthenogenetic teiid lizards *Aspidoscelis tessellatus* and *A*. *neomexicanus* [60] and in parthenogenetic *D*. *dahli*, *D*. *armeniaca*, and *D*. *unisexualis* [31]. *Darevskia rostombekowi* has two times lower clonal diversity than *D*. *dahli* [43]. Although data are limited, this suggests that either *D*. *rostombekowi* may have a more recent origin than *D*. *dahli*, or it suffered a severe bottleneck.

Some cytogenetic and molecular characteristics of the genome of hybrid species differ from those of the bisexual species due to disturbance in the segregation of chromosomes. This results in cytogenetic variability, different genomic activity and mutations in some loci [14,31,42,61]. Karyotypic instability of hybrid genomes may lead to the genetic diversity of parthenogenetic species and the appearance of chromosomal mutations [36]. Such mutations may govern the formation of new clones and/or geographically isolated chromosomal races. Our analyses cannot rule out the possibility that the isolated population of *D*. *rostombekowi* inhabiting the coast of Lake Sevan constitutes a chromosomal race and/or a clone that emerged because of chromosomal mutations occurring in the course of karyological evolution.

In summary, our analyses discover multiclonal genetic structure in the parthenogenetic lizard *D*. *rostombekowi*. Future studies may determine whether the clonal diversity owes to a single or multiple interspecific hybridization events, and identify the role mutations play in altering the initial clone(s).

## Supporting information

S1 Fig Alignment of Du281 allelic sequences containing microsatellites and neighboring regions, of parthenogenetic *D*. *rostombekowi* and parental *D*. *portschinskii* and *D*. *raddei*. *The number of alleles is shown in square brackets.
(TIF)

S1 Table Allelic variations of microsatellite containing loci in the lizard species *D*. *rostombekowi*, *D*. *raddei* and *D*. *portschinskii*. (DOCX)
