# Supplementary figures and images for: The origin of multiple clones in the parthenogenetic lizard species Darevskia rostombekowi
Source: PLoS One. 2017 Sep 20;12(9):e0185161. doi: 10.1371/journal.pone.0185161 (PMC5607197; doi:10.1371/journal.pone.0185161)

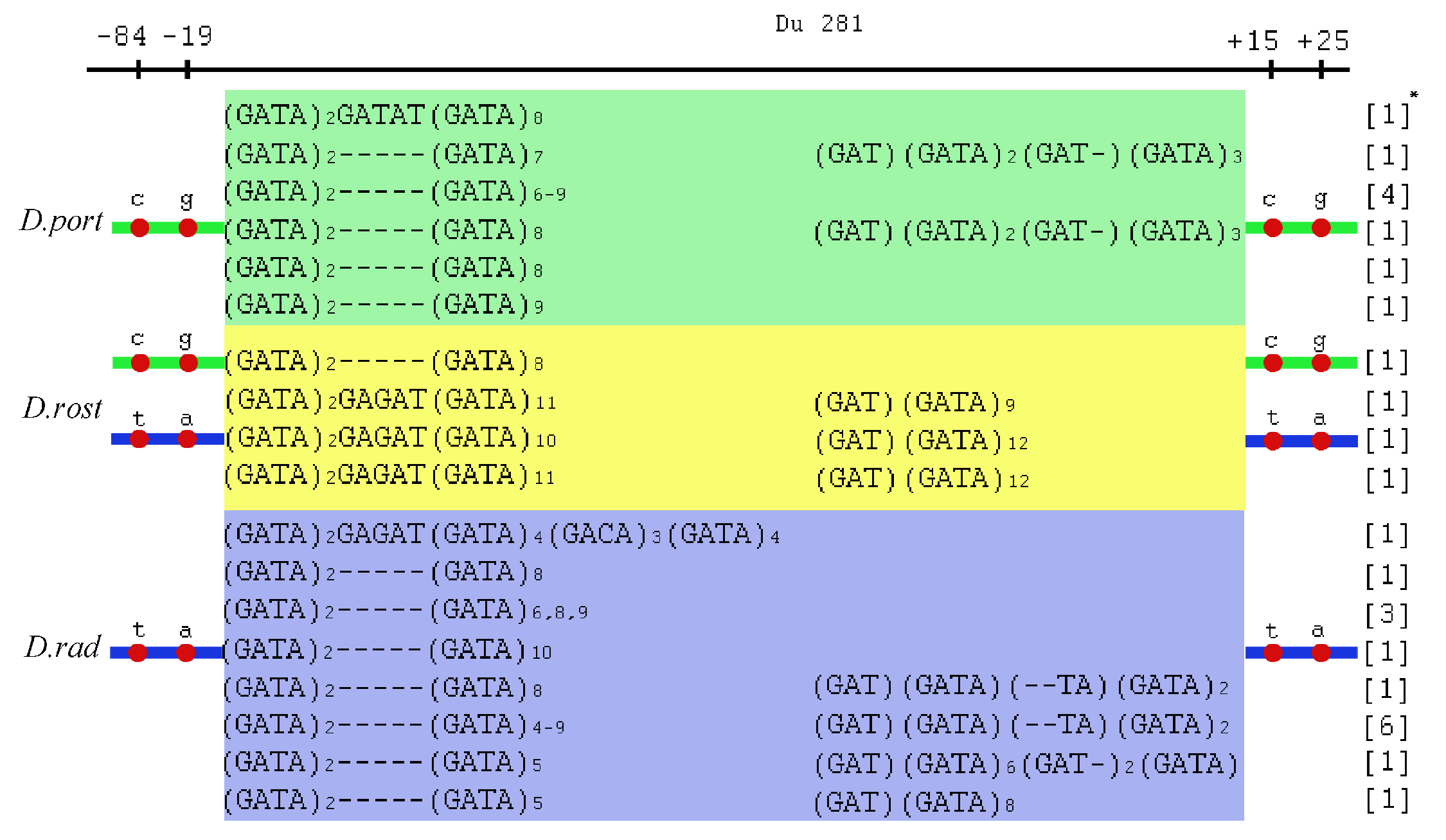

Supplement: S1 Fig — *The number of alleles is shown in square brackets. (TIF) [file pone.0185161.s001.tif]
