# Supplementary material for: The origin of multiple clones in the parthenogenetic lizard species Darevskia rostombekowi
Source: PLoS One. 2017 Sep 20;12(9):e0185161. doi: 10.1371/journal.pone.0185161 (PMC5607197; doi:10.1371/journal.pone.0185161)
Supplement: S1 Table — (DOCX) [file pone.0185161.s002.docx]

**S1 Table. Allelic variations of microsatellite containing loci in lizard species *D. rostombekowi, D. raddei* and *D. portschinskii.***

| **Allelic variant** | **Size (bp)** | **Structure of microsatellite cluster** | **SNV**  **(nucleotide position, N)** |
| --- | --- | --- | --- |
| **Du215(rost)1** | 256 | 5’ (GATA)_10_(GCAA)_11_ 3’ | **C (–19)** |
| **Du215(rost)2** | 252 | 5’ (GATA)_9_(GCAA)_11_ 3’ | **C (–19)** |
| **Du215(rost)3** | 250 | 5’ (GATA)_9_(GCAA)_10_ 3’ | TT (–98/99) **C (–19)** |
| **Du215(rost)4** | 248 | 5’ (GATA)_8_(GCAA)_11_ 3’ | **C (–19)** |
| **Du215(rost)5** | 227 | 5’ (GATA)_4_(GAT)(GATA)_7_(GCAA)_2_ 3’ | **C (–19)** |
| Du215(rad)1 | 256 | 5’ (GATA)_9_(GCAA)_12_ 3’ | C (–19) |
| Du215(rad)2 | 242 | 5’ (GATA)_8_GAT(GATA)_3_GATTGAT(GATA)_3_(GCAA)_2_ 3’ | T (–19) |
| Du215(rad)3 | 239 | 5’ (GATA)_4_GAT(GATA)_10_(GCAA)_2_ 3’ | C (–19) |
| Du215(rad)4 | 239 | 5’ GATT(GATA)_9_GAT(GATA)_4_(GCAA)_2_ 3’ | C (–19) |
| Du215(rad)5 | 235 | 5’ (GATA)_4_GAT(GATA)_9_(GCAA)_2_ 3’ | C (–19) |
| Du215(rad)6 | 235 | 5’ (GATA)_5_GAT(GATA)_8_(GCAA)_2_ 3’ | T (–19) |
| Du215(rad)7 | 235 | 5’ GATAGACA(GATA)_5_GAT(GATA)_6_(GCAA)_2_ 3’ | C (–19) |
| Du215(rad)8 | 235 | 5’ (GATA)_7_GAT(GATA)_6_(GCAA)_2_ 3’ | C (–19) |
| Du215(rad)9 | 231 | 5’ (GATA)_4_GAT(GATA)_8_(GCAA)_2_ 3’ | C (–19) |
| Du215(rad)10 | 231 | 5’ (GATA)_4_GAT(GATA)_8_(GCAA)_2_ 3’ | T (–19) |
| Du215(rad)11 | 230 | 5’ (GAT)_2_(GATA)_4_GAT(GATA)_7_(GCAA)_2_ 3’ | T (–19) |
| Du215(rad)12 | 227 | 5’ (GATA)_4_GAT(GATA)_7_(GCAA)_2_ 3’ | C (–19) |
| Du215(rad)13 | 227 | 5’ (GATA)_4_GAT(GATA)_7_(GCAA)_2_ 3’ | T (–19) |
| Du215(rad)14 | 227 | 5’ GATT(GATA)_6_GAT(GATA)_4_(GCAA)_2_ 3’ | C (–19) |
| Du215(rad)15 | 226 | 5’ (GATA)_4_GAT(GATA)_2_GAT(GATA)_4_(GCAA)_2_ 3’ | T (–19) |
| Du215(rad)16 | 223 | 5’ (GATA)_4_GAT(GATA)_6_(GCAA)_2_ 3’ | T (–19) |
| Du215(rad)17 | 223 | 5’ (GATA)_4_GAT(GATA)_6_(GCAA)_2_ 3’ | C (–19) |
| Du215(rad)18 | 219 | 5’ (GATA)_4_GAT(GATA)_5_(GCAA)_2_ 3’ | C (–19) |
| Du215(rad)19 | 215 | 5’ (GATA)_4_GAT(GATA)_4_(GCAA)_2_ 3’ | C (–19) |
| Du215(port)1 | 268 | 5’ (GATA)_12_(GCAA)_12_ 3’ | C (–19) |
| Du215(port)2 | 264 | 5’ (GATA)_11_(GCAA)_12_ 3’ | C (–19) |
| Du215(port)3 | 264 | 5’ (GATA)_10_(GCAA)_13_ 3’ | C (–19) |
| Du215(port)4 | 260 | 5’ (GATA)_10_(GCAA)_12_ 3’ | C (–19) |
| Du215(port)5 | 256 | 5’ (GATA)_9_(GCAA)_12_ 3’ | C (–19) |
| Du215(port)6 | 256 | 5’ (GATA)_11_(GCAA)_10_ 3’ | C (–19) |
| Du215(port)7 | 256 | 5’ (GATA)_10_(GCAA)_11_ 3’ | C (–19) |
| Du215(port)8 | 252 | 5’ (GATA)_10_(GCAA)_10_ 3’ | C (–19) |
| Du215(port)9 | 248 | 5’ (GATA)_10_(GCAA)_9_ 3’ | C (–19) |
| Du215(port)10 | 248 | 5’ (GATA)_9_(GCAA)_10_ 3’ | C (–19) |
| Du215(port)11 | 244 | 5’ (GATA)_10_(GCAA)_8_ 3’ | C (–19) |
| Du215(port)12 | 244 | 5’ (GATA)_11_(GCAA)_7_ 3’ | C (–19) |
| Du215(port)13 | 240 | 5’ (GATA)_9_(GCAA)_8_ 3’ | C (–19) |
| Du215(port)14 | 236 | 5’ (GATA)_9_(GCAA)_7_ 3’ | C (–19) |
| Du215(port)15 | 235 | 5’ (GATA)GACA(GATA)_5_GAT(GATA)_6_(GCAA)_2_ 3’ | C (–19) |
| Du215(port)16 | 228 | 5’ (GATA)_10_(GCAA)_4_ 3’ | C (–19) |
| Du215(port)17 | 228 | 5’ (GATA)_9_(GCAA)_5_ 3 | C (–19) |
| Du215(port)18 | 227 | 5’ (GATA)_4_GAT(GATA)_7_(GCAA)_2_ 3’ | C (–19) |
| Du215(port)19 | 224 | 5’ (GATA)_10_(GCAA)_3_ 3’ | C (–19) |
| Du215(port)20 | 220 | 5’ (GATA)_9_(GCAA)_3_ 3’ | C (–19) |
| Du215(port)21 | 216 | 5’ (GATA)_9_(GCAA)_2_ 3’ | C (–19) |
|  | | | |
| **Du281(rost)1** | 265 | 5’ (GATA)_2_(GAGAT)(GATA)_11_(GAT)(GATA)_12_ 3’ | T (–84), A (–19), T (+15), A (+25) **T (–21), А (+11)** |
| **Du281(rost)2** | 261 | 5’ (GATA)_2_(GAGAT)(GATA)_10_(GAT)(GATA)_12_ 3’ | T (–84), A (–19), T (+15), A (+25) **T (–21), А (+11)** |
| **Du281(rost)3** | 253 | 5’ (GATA)_2_(GAGAT)(GATA)_11_(GAT)(GATA)_9_ 3’ | T (–84), A (–19), T (+15), A (+25) **T (–21), А (+11)** |
| **Du281(rost)4** | 191 | 5’ (GATA)_10_ 3’ | C (–84), G (–19), C (+15), G (+25) **T (–21), А (+11)** |
| Du281(rad)1 | 226 | 5’ (GATA)_7_ GAT(GATA)_7_(GAT)_2_GATA 3’ | T (–21), А (+11) T (–84), A (–19), T (+15), G (+25) |
| Du281(rad)2 | 220 | 5’ (GATA)_7_ GAT(GATA)_8_ 3’ | T (–21), А (+11) T (–84), A (–19), T (+15), G (+25) |
| Du281(rad)3 | 214 | 5’ (GATA)_2_GAGAT(GATA)_4_(GACA)_3_(GATA)_4_ 3’ | T (–21), А (+11) T (–84), A (–19), T (+15), G (+25) |
| Du281(rad)4 | 208 | 5’ (GATA)_11_GATGATA 3’ | T (–21), А (+11) T (–84), A (–19), T (+15), G (+25) |
| Du281(rad)5 | 208 | 5’ (GATA)_10_GATGATATA(GATA)_2_ 3’ | T (–21), А (+11) T (–84), A (–19), T (+15), G (+25) |
| Du281(rad)6 | 205 | 5’ (GATA)_12_TAGATA 3’ | T (–21), А (+11) T (–84), A (–19), T (+15), G (+25) |
| Du281(rad)7 | 204 | 5’ (GATA)_10_GATGATA 3’ | T (–21), А (+11) T (–84), A (–19), T (+15), G (+25) |
| Du281(rad)8 | 200 | 5’ (GATA)_9_GATGATA 3’ | T (–21), А (+11) T (–84), A (–19), T (+15), G (+25) |
| Du281(rad)9 | 197 | 5’ (GATA)_10_TAGATA 3’ | T (–21), А (+11) T (–84), A (–19), T (+15), G (+25) |
| Du281(rad)10 | 196 | 5’ (GATA)_8_GATGATA 3’ | T (–21), А (+11) T (–84), A (–19), T (+15), G (+25) |
| Du281(rad)11 | 196 | 5’ (GATA)_4_GAAA (GATA)_3_GATGATA 3’ | T (–21), А (+11) T (–84), A (–19), T (+15), G (+25) |
| Du281(rad)12 | 195 | 5’ (GATA)_11_ 3’ | T (–21), А (+11) T (–84), A (–19), T (+15), G (+25) |
| Du281(rad)13 | 192 | 5’ (GATA)_7_ GATGATA 3’ | T (–21), А (+11) T (–84), A (–19), T (+15), G (+25) |
| Du281(rad)14 | 191 | 5’ (GATA)_10_ 3’ | T (–21), А (+11) T (–84), A (–19), T (+15), G (+25) |
| Du281(rad)15 | 188 | 5’ (GATA)_6_ GATGATA 3’ | T (–21), А (+11) T (–84), A (–19), T (+15), G (+25) |
| Du281(rad)16 | 183 | 5’ (GATA)_8_ 3’ | T (–21), А (+11) T (–84), A (–19), T (+15), G (+25) |
| Du281(port)1 | 217 | 5’ (GATA)_10_GAT(GATA)_2_GAT(GATA)_3_ 3’ | T (–21), С (+11) C (–84), G (–19), C (+15), G (+25) |
| Du281(port)2 | 213 | 5’ (GATA)_9_GAT(GATA)_2_GAT(GATA)_3_ 3’ | А (–21), С (+11) C (–84), G (–19), C (+15), G (+25) |
| Du281(port)3 | 195 | 5’ (GATA)_2_GATAT(GATA)_8_ 3’ | T (–21), А (+11) C (–84), G (–19), C (+15), G (+25) |
| Du281(port)4 | 195 | 5’ (GATA)_11_ 3’ | T (–21), А (+11) C (–84), G (–19), C (+15), G (+25) |
| Du281(port)5 | 195 | 5’ (GATA)_11_ 3’ | А (–21), А (+11) C (–84), G (–19), C (+15), G (+25) |
| Du281(port)6 | 191 | 5’ (GATA)_10_ 3’ | T (–21), А (+11) C (–84), G (–19), C (+15), G (+25) |
| Du281(port)7 | 191 | 5’ (GATA)_10_ 3’ | А (–21), А (+11) C (–84), G (–19), C (+15), G (+25) |
| Du281(port)8 | 187 | 5’ (GATA)_9_ 3’ | А (–21), А (+11) C (–84), G (–19), C (+15), G (+25) |
| Du281(port)9 | 183 | 5’ (GATA)_8_ 3’ | А (–21), А (+11) C (–84), G (–19), C (+15), G (+25) |
|  | | | |
| **Du323(rost)1** | 195 | 5’ (AC)_6_...(GATA)_6_(GAT)(GATA)_2_ 3’ | **C (–23), T (+39)** |
| **Du323(rost)2** | 184 | 5’ (AC)_4_…(GATA)(GGT)(GATA)_3_(GAT)(GATA) 3’ | **A (–23), C (+39)** |
| Du323(rad)1 | 184 | 5’ (AC)_4_...GATAGGT(GATA)_3_GATGATA 3’ | A (–23); C (+39) |
| Du323(rad)2 | 180 | 5’ (AC)_4_...GATAGGT(GATA)_2_GATGATA 3’ | A (–23); T (+39) |
| Du323(port)1 | 215 | 5’ (AC)_6_...(GATA)_11_GAT(GATA)_2_ 3’ | C (–23), T (+39) |
| Du323(port)2 | 211 | 5’ (AC)_6_...(GATA)_10_GAT(GATA)_2_ 3’ | C (–23), T (+39) |
| Du323(port)3 | 203 | 5’(AC)_6_...(GATA)_8_GAT(GATA)_2_ 3’ | C (–23), T (+39) |
| Du323(port)4 | 199 | 5’ (AC)_6_...(GATA)_7_GAT(GATA)_2_ 3’ | C (–23), T (+39) |
| Du323(port)5 | 195 | 5’ (AC)_6_...(GATA)_6_GAT(GATA)_2_ 3’ | C (–23), T (+39) |
| Du323(port)6 | 191 | 5’ (AC)_6_...(GATA)_5_GAT(GATA)_2_ 3’ | C (–23), T (+39) |
| Du323(port)7 | 187 | 5’ (AC)_6_...(GATA)_4_GAT(GATA)_2_ 3’ | C (–23), T (+39) |
| Du323(port)8 | 179 | 5’ (AC)_6_...(GATA)_2_GAT(GATA)_2_ 3’ | C (–23), T (+39) |
